# A randomized control trial to test a peer support group approach for reducing social isolation and depression among female Mexican immigrants

**DOI:** 10.1186/s12889-020-09867-z

**Published:** 2021-01-11

**Authors:** Janet Page-Reeves, Cristina Murray-Krezan, Lidia Regino, Jackie Perez, Molly Bleecker, Daniel Perez, Bill Wagner, Susan Tigert, Elaine L. Bearer, Cathleen E. Willging

**Affiliations:** 1 University of New Mexico, Albuquerque, New Mexico, USA; 2 Centro Sávila, Albuquerque, New Mexico, USA; 3 Pacific Institute for Research & Evaluation, Albuquerque, New Mexico, USA

**Keywords:** Social isolation, Depression, Peer support group, Women, Mexican immigrant

## Abstract

**Background:**

Female Mexican Immigrants (FMIs) experience high rates of depression compared with other populations. For this population, depression is often exacerbated by social isolation associated with the experience of immigration.

Aim 1. To measure whether a culturally situated peer group intervention will reduce depression and stress associated with the experience of immigration.

Aim 2. To test whether an intervention using a “women’s funds of knowledge” approach results in improved resilience, knowledge and empowerment.

Aim 3. To investigate whether a culturally situated peer group intervention using a women’s funds of knowledge approach can give participants a sense and experience of social and physical connection (“emplacement”) that is lost in the process of immigration.

**Methods:**

This mixed-methods study will implement “*Tertulias*” (“conversational gatherings” in Spanish), a peer support group intervention designed to improve health outcomes for FMI participants in Albuquerque, New Mexico. We will document results of the intervention on our primary hypotheses of a decrease in depression, and increases in resilience and social support, as well as on our secondary hypotheses of decreased stress (including testing of hair cortisol as a biomarker for chronic stress), and an increase in social connectedness and positive assessment of knowledge and empowerment.

**Discussion:**

This project will address mental health disparities in an underserved population that experiences high rates of social isolation. Successful completion of this project will demonstrate that health challenges that may appear too complex and too hard to address can be using a multi-level, holistic approach. Our use of hair samples to test for the 3-month average levels of systemic cortisol will contribute to the literature on an emerging biomarker for analyzing chronic stress.

**Trial registration:**

This study was registered with ClinicalTrials.gov on 2/3/20, Identifier #NCT04254198.

**Supplementary Information:**

The online version contains supplementary material available at 10.1186/s12889-020-09867-z.

## Background

Social Isolation is a critical health risk factor affecting millions of people in the U.S. and, disturbingly, has been shown to have an even larger social impact [1, 2]. While most people would not think of social isolation as a “health” issue per se, as a chronic stressor, it is a well-established health risk factor [3] associated with the experience of depression, loneliness, and other mental health problems [4–8], increased risk for the development of chronic disease [9–12], and even mortality [13–18].

Despite evidence that social isolation is both a major health risk factor and that it is increasing, it continues to be under-assessed and under-addressed [19]. There is a lack of consensus on how to screen for risk of social isolation [20], and few studies have attempted to document prevalence beyond narrow populations (such as older adults [21] or individuals with a chronic health condition [22, 23])**.** Most reviews of social isolation as a health risk suggest that actual prevalence in the U.S. is much higher than has been documented [24]. Because social isolation is caused by a complex concatenation of individual, family, social, and structural factors, any solution must go beyond individual treatment. Therefore, it is difficult to design an intervention that is functionally viable or economically sustainable in a clinic setting. The need, then, is determining what type of intervention can be developed given that few successful or sustainable examples have been documented [17].

Cacioppo & colleagues describe how a lack of social relationships can become “toxic” [25], with severe, negative, cascading impacts on individual health and family wellbeing [17, 19]. However, the challenge of dealing with “toxic” social isolation is disproportionately greater for immigrants and contributes to mental and physical health disparities [26–28]. Immigrants leave behind meaningful social, emotional, and cultural connections in their home country. In the new setting, it is often difficult to create social relationships, feel socially, emotionally, and culturally connected to other people, or have knowledge regarding access to essential resources. Language barriers, social stigma, discrimination, and poverty [29]—the structural circumstances [24] of immigrants’ lives—exacerbate this dynamic. Increasingly, the migration experience itself is understood as a risk factor for clinically significant mental health problems [30]. For Mexican immigrants, despite a protective effect of immigration on health discussed as the “Hispanic paradox” [31] in which many first generation immigrants demonstrate better health than their U.S.-born counterparts, further cross-border research [32] that controls for demographic variables has revealed that Mexican immigrants have twice the risk (odds ratio of 1.8) for first onset of any depressive disorder than those in Mexico who do not immigrate. As such, recognizing social isolation and related depression as health disparities requires that we consider both the complex, multifactorial nature of how social isolation operates in the lives of immigrants through multiple “social determinant” pathways, as well as the multilevel impact on individuals, families, and the immigrant community. Merrill Singer [33] conceptualized how multiple streams of influence can come together to form a “syndemic” in which the synergistic interaction of social, environmental, economic, and political factors produce disease—often disproportionately affecting a particular group. The syndemic framework was explored in a recent special edition of The Lancet [34, 35] as a comprehensive approach for addressing health disparities. A syndemic framework is useful for thinking about how social isolation creates toxic health problems in immigrant communities and highlights the need for holistic solutions.

Women immigrants, in particular, are at high risk for social isolation [26, 27, 36–41]. For many first generation female Mexican immigrants (FMIs) from low-income households, the elements that have been identified as key protective factors [42–44] (culture and family) are eviscerated by social isolation. For FMIs, social isolation from the loss of extended family networks is often experienced as a form of cultural bereavement [26] that is emotionally traumatic [27–32, 39–45], leading to disproportionately high rates of depression. As a group, Latinas (Hispanic women) have higher rates of depression (14%) compared with non-Hispanic white women (7%) [42]. Yet troublingly, Latinas are less likely to receive mental health treatment (56% vs 72%) [46]. However, research has shown that for FMIs, while many recent immigrants report less depressive symptomology the closer they are to arrival in the U.S., the longer a FMI lives here, her risk for depression increases [42]. Moreover, the rate of depressive disorders among FMIs is twice that for male Mexican immigrants [41–46]. While it is challenging to find statistical data to describe an immigrant subgroup such as FMIs, a number of studies that disaggregate data or that have focused specifically on FMIs reveal alarming insights. A study with Latinas in South Florida, 95% of whom were immigrants including many FMIs, found that 38% of participants screened positive for depressive symptoms [47]. In a study of a nursing intervention with FMIs and their children, 36% of participants reported depression at or above cut-off for referral—higher than the national average of 10% for all Americans [48]. And in a study of Mexican immigrant patients at a health center in San Antonio, FMIs were three times more likely (32%) than non-Hispanic white women (10%) to present with depression [49].

In two of our prior community-engaged, NIH-funded research projects, we revealed the key nature of group interaction in the lives of Latina women, including FMIs. One of our projects [36–38] focused on women’s social networks and food insecurity in a historic Latino community, and our intervention involved project events (“fiestas”) where women learned to lead meetings and interacted with each other in a way that resulted in increased participant ability to adapt to new circumstances (resilience), increased recognition of personal assets (empowerment) and more social connectivity. Social interaction helped participants to see their own reality reflected in the lives of other women. Information presented and discussed at project events improved critical understanding of the things that influence people’s lives and increased women’s desire to become involved in the community.

In our other study [50], we worked with a FMI social isolation support group. For immigrant women, the realities of the immigration experience mean that the process of building relationships and creating or finding a social network of support is often extremely challenging [51]. We found that 100% of the FMI participants reported that their experience in the support group had been helpful or even transformational in their lives in relation to forming social relationships and friendships, and increasing their sense of social connectedness by making them feel as if they are not alone. Listening to others, and sharing experiences, feelings, ideas, and information helped them understand their own problems and the problems of others with a new lens. Participation in the group also reduced depression and relieved stress and anxiety. And, participants reported that participation in the group was empowering to them as women. For FMIs then, social interaction plays a critical role in assisting them to rebuild their lives, redefine or reclaim their identity(s), and maintain health through shared knowledge and access to resources [52–54]. The group experience in our project was key in creating a mechanism for positive social engagement.

**Fig. 1:**
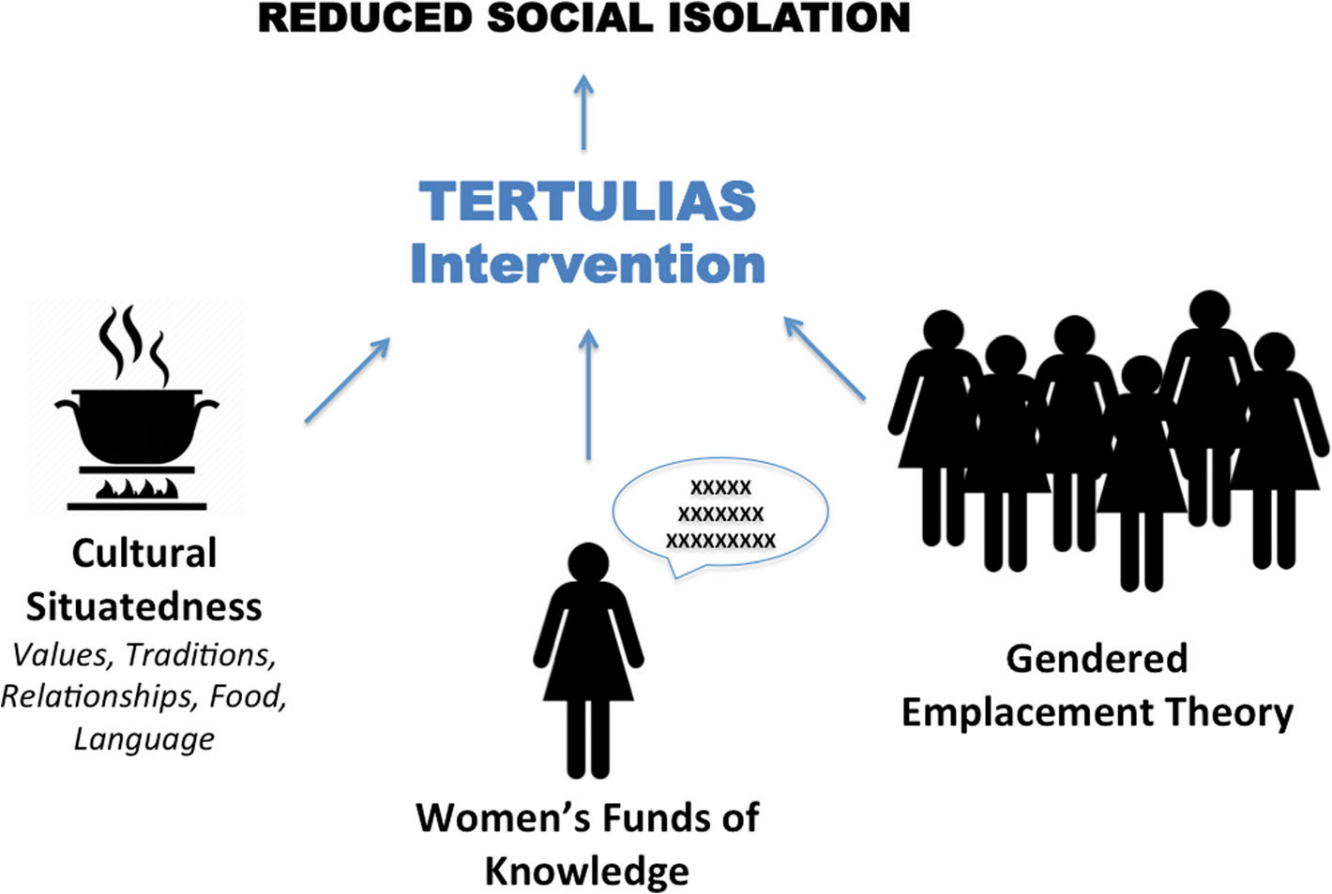
Theoretical architecture

To account for the syndemic complexity associated with understanding the interconnectedness of the domains of inquiry for this study, our hypothesis and research design are based on a fusion of three theoretical frameworks [see Fig. 1]:

### Cultural and contextual situatedness

Trickett’s conceptualization of cultural and contextual situatedness [55–57] proposes integrating components of an intervention “into the local expression of culture as reflected in the multiple levels of the ecological context [55].” Rather than merely “tailoring” an intervention to target a specific context or population (*e.g.,* by offering curriculum materials in Spanish or using images of individuals who “look” Latino), Trickett emphasizes the need for an intervention to be “*situated*” to fit *synergistically* within broader community dynamics (*culture and context*). An intervention should find a cultural and contextual “fit,” but it should also be strategically designed to leverage dimensions of culture and context to enhance intended outcomes. Thus, culture becomes a vehicle for promoting positive outcomes.

### Funds of knowledge

Moll, Gonzalez, & colleagues’ notion of funds of knowledge [58–60] from the literature on learning and culture has implications for health intervention programming for FMIs. They postulate that women have “historically accumulated and culturally developed bodies of knowledge and skills” [58]. We propose that women bring these funds to their participation in health interventions and that the success of a health intervention is mediated by the extent to which program design positively leverages cultural values and accommodates the socio-economic circumstances of a population in a way that incorporates and values participants’ knowledge and experience (*funds of knowledge*), and creates synergy with the specific social dynamics that define participants’ everyday lives (*situated in participants’ culture and context*). This synergy creates a learning environment that promotes transformative personal and social change, and nurtures empowering individual psychosocial dynamics that increase resilience and improve health outcomes.

### Gendered emplacement theory

Gendered emplacement theory [61–64] informs our use of a structured dialogue peer support group as the foundational design of the intervention. A large body of literature has explored the significant ways place is connected to health and wellness [65] through multi-level environmental influences in which an individual is embedded—many of which are defined as the social determinants of health. However, social science researchers have identified that the ways that a person experiences and perceives a connection to a place is also key. This sense of “*emplacement*,” both spatial and social, plays an important role in personal wellbeing. Social connectedness is an essential component of this dynamic. Feminist scholars have expanded this concept through investigation of the gendered dimensions of “emplacement” in which women are *displaced* from historically male dominant spaces and therefore compelled to create alternative, protected spaces for feeling connected and engaged [61–64]. For obvious reasons, immigrants experience this sense of *displacement* in an inherently heightened way, which for women immigrants is doubly disorienting.

#### Aim 1

To measure whether a culturally situated peer group intervention will reduce depression and stress associated with the experience of immigration**.** *Question*: Does an intervention design that reproduces culturally important interactions, activities, and constructs lost through immigration result in decreased participant depression and stress? *Hypothesis*: Incorporating peer-to-peer social interaction and storytelling into the design of a nonclinical peer support group intervention will leverage positive aspects of participant culture and create an experiential context that will (a) decrease participant depression scores by at least 6.5 points more on the Center for Epidemiologic Studies Depression Scale (CES-D) as compared to controls (effect size Cohen’s *d* = 0.43), and (b) lower stress scores in participants more than in controls with *d* ≥ 0.5 as measured by the Perceived Stress Scale (PSS). We will also assess stress using a cutting-edge biological assessment of hair cortisol as a biomarker for chronic stress.

#### Aim 2

To test whether an intervention using a “women’s funds of knowledge” approach results in improved resilience, knowledge and empowerment. *Question*: Does an intervention design that encourages participants to share knowledge they developed through life experience and that values this knowledge as a form of expertise nurture protective factors (resilience and knowledge/empowerment) to help FMIs adapt to the immigration context and disrupt the mechanisms that produce health disparities? *Hypothesis***:** Incorporating, valuing and validating women’s knowledge and experience in the design of a peer support group intervention will improve participant capacity to adapt to the immigrant context and provide participants with empowering knowledge to deal with new situations. Intervention participants will have higher scores at 12 months and have a larger increase over time as compared to controls (*d* = 0.5) on the Connor-Davidson Resilience Scale-25 (CD-RISC 25). We will assess knowledge and empowerment at 12 months and expect to find high scores with the Trauma-Informed Practice (TIP) Scale (which is designed for post-use).

#### Aim 3

To investigate whether a culturally situated peer group intervention using a women’s funds of knowledge approach can give participants a sense and experience of social and physical connection (“emplacement”) that is lost in the process of immigration. *Question*: Can the proposed peer support group intervention recreate social and physical connections lost through immigration and strengthen participant social networks? *Hypothesis*: The peer group will create a culturally appropriate context for establishing interpersonal connections between group members and will give participants a sense of belonging within a social and contextual milieu. At study end, (a) experimental participants will have a marked increase in social support scores v.s. the control group using the Medical Outcomes Study Social Support Survey (MOS SSS) (*d* ≥ 0.5), and (b) stronger, more dense social connections as described by a social network analysis.

## Methods/design

This study is a randomized control trial (RCT) with open assignment to two groups—one group receiving the study intervention and the other with no intervention. This study will implement the “Tertulias” peer support group intervention [50] that we developed to improve health outcomes and reduce health disparities related to social isolation and depression for FMI participants in Albuquerque, New Mexico. We will build on and leverage our extensive preliminary research about women’s social networks [36, 37] using a multi-level, community-engaged approach. Our intervention uses an innovative, culturally and contextually situated peer support group design that we developed to improve health outcomes and reduce health disparities for FMI participants in Albuquerque, New Mexico [50].

We will use rigorous, mixed-method research to document results of the intervention on our primary hypotheses of a decrease in depression and increases in resilience and social support, as well as on our secondary hypotheses of decreased stress (including an innovative test of cortisol levels in hair as an objective biochemical indicator for degree of chronic stress), and increases in social connectedness and positive assessment of knowledge and empowerment gained through the Tertulias intervention.

We will test whether the intervention reduces social isolation as a mechanism for reducing depression by leveraging positive cultural dynamics and women’s funds of knowledge to nurture social connectedness, knowledge, and resiliency factors in the lives of participants in a transformative way.

The proposed study will be conducted in New Mexico by researchers at the University of New Mexico in Albuquerque. The Tertulias intervention groups will be conducted at the facilities of our partner organizations: The Hopkins Center/Centro Sávila and One Hope Centro de Vida Health Center. Both partners are nonprofit healthcare organizations, The Hopkins Center/Centro Sávila provides clinical behavioral and mental health counseling. One Hope Centro de Vida Health Center is a community-run health clinic.

This project focuses on FMIs. All participants will be female immigrants over the age of 18 who were born in Mexico, report household income below 250% of the Federal Poverty Level (FPL), speak Spanish fluently, and who are not incarcerated.

We will test the integration of our three theoretical frameworks in the Tertulias peer support group intervention. Peer support groups have been shown to be efficacious for a variety of contexts, populations, and health conditions [66–70]. For women immigrants, culturally based interaction with peers in protected spaces that promote personal empowerment is important for rebuilding lives, redefining or reclaiming identity(s), and knitting a strong social fabric to make women and their families healthy and resilient [36, 37, 51, 71–73]. Yet just bringing women together would not likely result in systematic change to improve health outcomes and reduce health disparities. As per our theoretical architecture, cultural and contextual dimensions of women’s lives need to be considered (*situated*), women’s own knowledge and experience (*funds of knowledge*) must be valued and validated, and women need to develop a feeling of personal connection with others and have a way to conceptually understand their lives within a larger social context (*emplacement*). In our preliminary research, we worked with women from the Latino community, including FMIs, to develop a novel “structured dialogue” approach for engaging small groups of participants in a way that integrates these three theoretical frameworks [36, 37, 50]. The structured dialogue approach upon which the Tertulias intervention is based involves three fundamental components:

### Peer-to-peer interaction

In both of our previous projects, participants shared stories, experiences, and ideas with each other. Through the group design of the intervention, participants expanded their social networks by making friends, helping each other, and learning about resources available in the community. From each other, they learned about, explored, discussed, and analyzed issues that they found to be of interest, they listened to ways that others in the group (women like themselves) confronted challenges and resolved problems, they developed their skills and knowledge for dealing with the situations that they experience, and they increased their capacity to deal with adversity with resilience.

### Bi-directional expert facilitation

In our prior research, a trained facilitator led bi-directional “structured dialogue” that allowed discussion to occur in an organic, open-ended manner**.** The facilitator built on themes emerging in the peer-to-peer discussion by presenting powerful life stories and analyses from other studies of women’s health, and themes that emerged from interviews with individual participants. The group considered stories and themes that were presented and discussed them in relation to their own lives and experience—weaving together their own experiences with those of other women, both inside and outside the group. Through this process, they developed an understanding of the broader forces that influence and structure their choices and practices on an everyday basis. We found that this approach engaged women in a way that decreased depression and other negative emotional states, improved their capacity to deal with socioeconomic conditions and relationships with intimate partners, and gave them a new way of thinking about their own lives and for understanding their community [36, 37, 50]. Women described these changes as personally empowering and transformative.

### Storytelling

Storytelling is a central component of the innovative structured dialogue group intervention that we have developed. Both of our prior projects with women used storytelling, an evidence-based strategy from research on empowerment among women that has been found to be particularly effective among women of color [74]. In our projects, the participants shared stories of their experiences, or the facilitator presented stories from other women. For women in our studies, the act of storytelling was evocative. They shared experiences, expressed feelings, and saw the connection between their own stories and stories of others. Women found participation in this process to be empowering, giving them information and tools to understand the root causes of disparities they recognize in their own lives. The group-centered learning was generative through the development of social connections, and the identification of both individual actions and group strategies to improve health. Structured dialogue helped women participants create their own narratives in a way that was healing and personally transformative (*empowering*). The peer-to-peer nature of the proposed structured dialogue group intervention will help participants “locate” and “ground” (*emplace*) themselves within a supportive social network. The critical social “literacy” that accrues from the structured dialogue design of our model will help women further “locate” and “ground” themselves in relation to shared stories and experiences—stories and experiences from others in the group and those presented by the facilitator—that tether participants’ lives to policy and structural relations of power through transformative learning, building resilience, and becoming empowered.

The primary outcome measures are changes from baseline to 12 months in:

1. Depression as measured by the Center for Epidemiologic Studies Depression Scale (CES-D) [75, 76]
2. Resilience as measured by the Connor-Davidson Resilience Scale-25 (CD-RISC 25) [77]
3. Social support as measured by The Social Support Scale (MOS-SSS) [78, 79]

The secondary outcome measures are:

1. Changes in stress from baseline to 12 months as measured by:
  - The Perceived Stress Scale-14 (PSS-14) [80]
  - Hair cortisol levels [81–86]
2. Knowledge and empowerment in the intervention arm only at 12 months, measured by the Trauma-Informed Practice (TIP) scale [87, 88]
3. Changes from baseline to 12 months in social connectedness as measured by Social Network Analysis [77, 79]

**Fig. 2:**
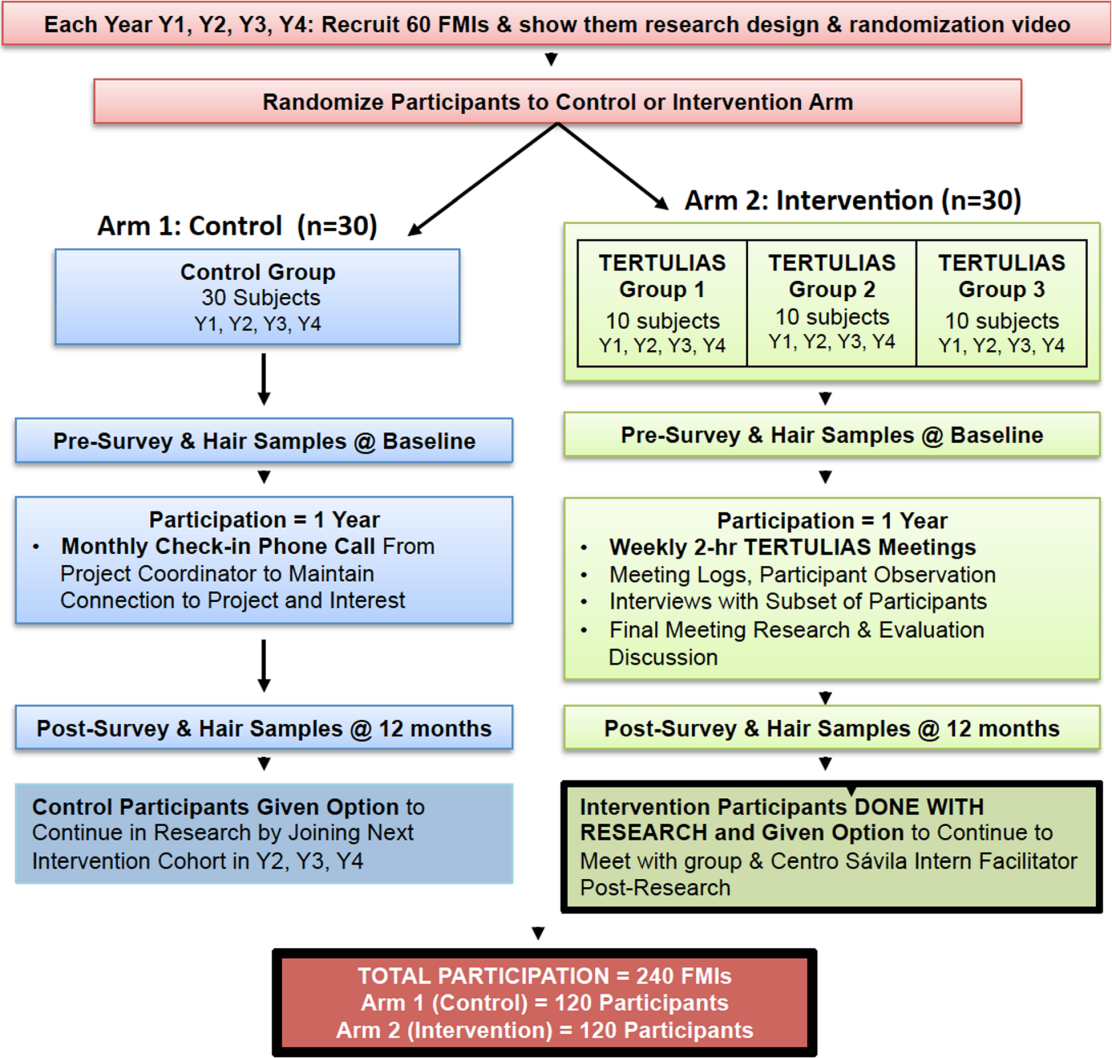
Recruitment table & participant timeline

We will recruit 240 participants (*N*= 240). We will randomize participants into one of two arms (Fig. 2):

#### Arm 1 (*n* = 120)

Control participants will be recruited in four 12-month cohorts of 30 each, with 20 at one site and 10 at the other.

#### Arm 2 (*n* = 120)

Intervention participants will be recruited in four 12-month cohorts of 30 each. Each cohort will be divided into three groups of 10 women, with 2 groups at one site and 1 group at the other. Over the four cohort cycles, we will have a total of 12 groups. Sample size estimates accommodate 33% attrition**.**

With the expectation that 100 participants will complete the study in each arm, the following minimum hypothesized changes in outcome measures will be detected with > 80% power, assuming type I error = 0.017 for three primary outcome measures (one in each Aim):

1. An 8-point decrease in depression as measured by the CES-D for the Intervention arm, from baseline to 12 months, with at least a 6.5-point greater decrease than control participants (between arms effect size Cohen’s *d* = 0.43 with SD = 15), as shown to be feasible in prior work [89, 90]
2. Cohen’s *d* effect size ≥0.33 comparing arms on the changes over time of the CD-RISC scores, indicating increased resiliency in the intervention arm as compared to the control arm [91–93]
3. Decreased social isolation as measured by the MOS-SSS in the intervention arm as compared to the control arm over time with Cohen’s *d* ≥ 0.5 (SD =16) [94–96]

We will recruit a total of 240 FMI participants through our two partner agencies that both have a large Mexican immigrant clientele. We will document successful recruitment strategies**.** We will track methods used to identify potential participants at each site, number approached for interest, number who are interested/not interested, number who are screened for eligibility, and number of eligible participants who agree/decline to enter the study.

When we designed the project, our plan was to hold an in-person interest meeting in Spanish with all potential participants where we would show them a PowerPoint presentation explaining the project and a brief video [97] that we produced about the concept of randomization (both the PowerPoint presentation and the video are in Spanish). The video has FMI presenters describing the scientific and social contribution that this study will make, how randomization works, and why randomization is important, to help participants understand why we have structured the project to have two arms.

However, because of required safety restrictions related to the COVID-19 pandemic, we amended our approach to be able to hold the meeting remotely by Zoom. We created a user-friendly Zoom instruction guide in English and Spanish, and the project coordinators leading the recruitment process will contact participants individually to ensure that they have a device and internet service they can use to connect to the Zoom meeting and that they become familiar with how to use Zoom. We also decided that instead of having one large interest meeting, we will hold a series of smaller meetings in order to ensure that potential participants have sufficient opportunity to ask questions.

After viewing the presentation and the video, and asking any questions, those interested in participating will be randomized using a block randomization design to allocate 50% of participants to the control group and 50% to the intervention. Random block sizes of 2, 4, and 5 will be implemented to ensure balance between arms. The randomized allocation sequence will be generated in R version 3.6 [98] and implemented in REDCap [99], the electronic data capture system for this study, by the biostatistician (Murray-Krezan). Participants will be assigned to one of two arms per the randomized allocation schedule accessed in REDCap. Intervention group participants will be offered three group meeting options to accommodate participants’ schedules related to day/time and location considerations, but the three options will all be conducted using the same structure and format. Baseline data collection appointments will be scheduled to occur within 4 weeks of when Zoom project interest meetings are held prior to beginning the Tertulias intervention in each cohort. All participants will provide signed informed consent (in English or Spanish, depending on participant preference) at the time of their baseline appointment prior to data collection.

During the project interest meetings conducted via Zoom, each woman will choose a number. They will be shown a grid with all available numbers in squares. Each woman will be asked to choose a number. When the number is chosen, the square with the number will be changed to a black background and the Project Coordinator (D. Perez) will keep track of the numbers off screen. After all participants on in the meeting have chosen a number, the Project Coordinator will visually present the participant’s name and reveal the arm to which the participant has been assigned.

#### Arm 1: control

Using a modified Attention Control Placebo (ACP) design [100] to reduce attrition over the 12 months, control arm participants will receive a bimonthly phone call from our project coordinator to check in with them, make sure their contact information is correct, and let them know that the study is continuing.

#### Arm 2: intervention

We will conduct Tertulias structured dialogue groups using the model we developed and tested through our preliminary research. Each group will have 10 women and will meet weekly for 2 h over a 12-month period. Group meetings will be conducted in Spanish, led by a team of two FMI facilitators using our structured dialogue approach. Because of required safety restrictions related to the COVID-19 pandemic, our original intention to hold these groups in-person was amended to hold the groups remotely by Zoom. We will work further with each Arm 2 participant to ensure that they know how to use Zoom and have electronic access. Because we recognize that a 2-h weekly meeting will use a significant amount of data, we will provide each Arm 2 participant with $30/month to support them to have a data plan sufficient to provide access for the meetings. Because we also recognize that the participants may be staying at home with children or other family members in a small space, we will also provide each group participant with a set of “earbud” style audio-phones to be able to provide privacy for the group from others who may be present in the room with the participant during the meetings.

We will gather data through in-person data collection appointments at baseline and 12 months. Data collection appointments will be in-person in order to gather hair samples unless there are safety concerns (e.g., COVID-19 illness in the participant’s home, a COVID-19-positive test within the past 2 weeks, fever, cough, travel outside the state within the past 14 days, etc.). In the case of participants with these concerns, we will conduct the survey by phone and gather the hair sample at another time if resolution of the concern is able to be made within the timeframe of a week after starting the meetings. All in-person data collection appointments will be conducted using COVID-19 safety procedures involving screening questions, temperature check, hand sanitizer, and face masks for both the data collector and the participant. Additionally, data collectors will also wear face shields, gloves, and shoe covers.

### Surveys

We will administer a pre/post survey to all participants at baseline prior to the first group session and at 12 months. The survey will combine demographic questions with questions from validated instruments to measure domains of interest (Depression [75, 76], Stress [80], Resilience and Adaptability [77], and Social Support [78, 79] plus questions documenting the participant’s social network to measure social connectedness [78, 79] to inform a social network analysis. In addition to the pre/post survey all participants will receive at 12 months, Arm 1 (*control*) participants will be asked to answer questions about interest in participating in an intervention group, and Arm 2 (*intervention*) participants will be asked a series of questions designed to evaluate their participation in and perspective on the Tertulias intervention, including knowledge and empowerment [87, 88]. At each time point, the survey will be administered orally in Spanish. We have developed a protocol and have professional team members to assist any participant (control or intervention) identified as needing immediate medical or counseling services (see below)**.**

### Hair cortisol bio-measure of chronic stress

Health disparities research has matured through the application of the social determinants of health framework. However, the need to move beyond merely understanding social determinants to action-oriented intervention research is increasingly recognized. Identifying biomarkers to support this work is imperative. Therefore, as part of this project, we will gather hair samples to test for cortisol, a biomarker of stress. We know that stressful experience influences physical and psychological health [101], and we now understand that stress can accumulate or become toxic, ultimately influencing the biology of the human body, including the hormones, cortisol and adrenalin, and the immune system. Such chronic toxic stress is an emerging construct in health research [102–104]. However, measuring stress biochemically in the context of research is challenging. Many studies (the proposed study included) use psychosocial instruments that rely on self-report or recall. While often revealing valuable information, data from these instruments can be limited by respondent subjectivity or reporting bias [105]. Hence there is a critical need to develop an objective biomarker that can be used for unbaised measurement of chronic stress, such as altered hormone levels that the experience of stress provokes [106]. In the context of this study, advances in studying hair cortisol, an emerging validated biomarker for chronic stress [82, 107] offer exciting opportunities**.** Cortisol levels are altered in people with chronic stress. Cortisol has traditionally been measured in blood, saliva, or urine by a variety of clinical testing approaches [86]. Because of the diurnal variation in cortisol levels, these measures are unreliable. Furthermore, measures of bodily fluids offer a snapshot of cortisol levels at any particular moment that can have valuable clinical uses, but fail to reveal the bigger picture of chronicity. Unlike bodily fluids, hair provides a long-term read-out of cortisol levels over time. Typically hair grows by 1 cm per month. Therefore, cortisol extracted from 3 cm of hair detects the average cortisol levels in that person over the preceding 3 months. This innovative, non-invasive bio-measure of long-term or chronic stress is emerging with applications to a range of questions, including stress and diabetes, stress and poverty, and stress and child maltreatment**.** In recent literature, measurement of cortisol levels in hair has proven useful to determine the long-term effects of stress [86, 108–112], including in relation to depression [113–115]. Previously, there has been no good assay to determine this effect. Now, with the ability to measure average cortisol over a span of time, more complex relationships will be identified and analyzed [82, 116]**.** This line of inquiry is an major advance in revealing the often subtle ways that dysregulation of cortisol levels is implicated in the risk of disease and negative health outcomes [113]. We will gather hair samples from all participants at baseline and 12 months to measure average levels of circulating cortisol as a biological marker for chronic stress [81–86, 117]. The data collectors will use scissors to obtain a pencil-width (at least 3 cm long) of hair from the crown of the participant’s head as described [85, 118]. Hair will be stored in pre-prepared foil packets placed inside plastic bags to maintain the orientation of the head-end of the hair sample. Cortisol will be extracted from 3 cm of hair measured from the scalp as described elsewhere [81–86, 117, 118]. Hair will be pulverized in a Retsch Mixer Mill Type MM 400100-240 V 50/60HZ and extracted in methanol overnight, dried and re-suspended in buffer. Cortisol concentration will be measured by the Salimetrics expanded range, high sensitivity enzyme immunoassay (item 1–3002, Salimetrics, State College, PA). Cortisol levels will be compared across our dataset, and mean ± standard deviation determined. Levels ±2 S.D. from the mean will be considered elevated or depressed. Change in levels between time points (before and after the intervention) will be compared. Pregnancy (determined by a question on the survey) will be considered in our analysis, as well as hair products or steroid-containing topical lotions and drug therapies.

### Participant observation and meeting logs

We will conduct participant observation of Tertulias group sessions. An experienced observer will attend Tertulias group meetings on a rotating basis and keep an observational log on a laptop computer. Facilitators will maintain weekly group meeting logs to document group dynamics of interest. Observation notes and logs will serve as objective measures of dimensions of women’s participation and aspects of the group dynamic of interest in this study.

### Policy log

We will document multilevel impacts**.** In our previous work, we documented that Tertulias have multilevel impact on individual women, on women’s family relationships and families, and on the group. In addition, we recognize that some of the factors influencing women’s lives in relation to social isolation may be larger structural, systems, or policy issues that are outside the control of individual women, their families, or the group that could be addressed or targeted for change in a future project. Facilitators and the participant observer will keep a policy component as part of their logs to document these types of issues identified from group discussions.

### Interviews

We will conduct interviews with a subset of participants (*n* = 24). In keeping with standards for systematic theoretical sampling in qualitative research [93, 119–121], interviewees will be identified by the facilitators as individuals likely to have something to contribute to our understanding of the group dynamic. Interviews will be conducted in Spanish or English, depending on the preference of the participant, and will last approximately one to two hours. Because of COVID safety restrictions, interviews will be conducted by Zoom when appropriate. We will use an ethnographically inspired approach to allow participants to drive the flow of the interview. Questions will be posed in a semi-structured format with some open-ended questions. The interviewer will use interviewee responses to formulate prompts and follow-up questions. When appropriate in the context of the interview and depending on the content of the interviewee’s narrative, concepts from preceding interviews and group session will be incorporated into prompts and follow-up questions to engage the interviewee in a discussion of ideas or issues emerging within the context of the research. Interviews will be audio-recorded and transcribed/ translated. Interviewees will receive a $50 merchandise card.

### Group discussion

We will use the final group session at the end of 12 months for each of the 12 groups to ask participants to discuss the intervention (*n* = 12), its impact on their lives, and the domains of interest related to health outcomes. The sessions will be audio-recorded, transcribed, and translated. Participants will receive a $50 merchandise card.

Consents and contact information will be assigned a study ID. The ID will be the only links between the participant and the data. These will be stored in separate locked cabinets in the office of the PI and with access only by the investigators. Contact information will be destroyed 5 years following the end of the study. Consent Forms and the list of those who provided oral consent will be kept for 3 years following the end of the project, at which time they will be destroyed.

### Data management

Participant demographics, hair sample testing results, and survey data will be entered into REDCap. Data from REDCap will be exported to STATA and stored on a secure, managed network share maintained by our university’s Health Sciences Library and Informatics Center’s IT services. Data from observational notes and meeting logs will be stored on the password-protected computer of the PI. Data from interviews and group meetings will be captured on an audio-recording device and transcribed. If in Spanish, transcripts will be translated into English for analysis. Following transcription/ translation, audio-recordings will be destroyed. Transcripts of interviews and focus groups will be identified by the participant ID. Electronic transcript files will be stored on secure UNM computers, accessible only to the researchers via their password-protected machines. Hard copy data will be stored in binders in the locked offices of the PI. De-identified project data will be kept for at least 5 years.

### Descriptive statistics

Descriptive statistics (e.g., means, standard deviations, medians, frequencies, and percentages) will be calculated to summarize characteristics of the study arms. Outcome measures will be assessed for departure from normality and if detected, remedial measures such as appropriate transformations or robust methods will be used for analyses. For the primary and secondary outcome measures, unadjusted mean changes over time will be calculated and compared between the study arms. Linear mixed models will be fitted to the measures with the primary independent variable of interest Study Arm. Covariates will include age, marital status, number of children, other family or close friend living in same location as participant, speaks English, employed, health status, and number of group sessions attended. Least squares mean estimates and their 95% confidence intervals (CIs) for the changes over time and for the difference between arms will be calculated for the primary and secondary outcome measures. Summary measures (means or medians and corresponding 95% CIs, as appropriate) will be computed for hair cortisol measures at each time point and the mean changes and 95% CIs over time in each arm will be calculated. Correlation and regression analyses will be performed to understand whether change in hair cortisol levels, as a marker for stress, are related to our primary and secondary outcome measures. Calculations of cortisol will take into account its trimodal distribution, where abnormal values may be higher or lower than normal. The extent of missing data will be examined, and the patterns of missing data will be assessed for randomness. Multiple imputation methods will be employed if appropriate. Statistical analyses will be performed in SAS 9.4 and R 3.6 or higher.

### Social Network Analysis (SNA)

Social network analysis (SNA) will involve three types of analyses: 1.) Baseline and post-survey individual networks, 2.) The level of inclusion of other study participants in individual social networks, 3.) Changes in each study participant’s awareness of, knowledge about, and likelihood of accessing resources. The results from these three types of analyses will be “mapped” using UCINET social network analysis software [122].

### Qualitative data analysis

Qualitative data analysis will use accepted qualitative analysis techniques to analyze observational, group, and policy logs, participant observer notes, transcripts from interviews and the final group session, and the stories, poetry, and art created by the participants as part of the group process**.** We will review these data using a rigorous, disciplined approach to create an empirical analysis according to Hammersley’s [123] criteria for qualitative research based on plausibility, credibility, and relevance. We will follow Gläser and Laudel’s [124] framework for theory-driven qualitative content analysis. We will review the transcripts and identified conceptual categories and patterns related to the domains of inquiry, extract data, and develop conceptual summaries. Following review and summary, we will code extracted data internally for systematic subthemes and their domains. We will use “constant comparison” [125] to explore interconnections between theme/sub-theme categories and make connections with concepts we identify in the literature by developing a holistic interpretation of the data. Input for the interpretation will also be provided by the Advisory Board. Based on this analysis we will further refine conceptual categories and patterns, and we will develop conceptual summaries of issues of interest. Using this refined analysis, we will conduct a secondary review of the literature to identify further ideas, concepts, and approaches to help us understand what we were seeing in the data, and we will identify ways that data from this study and our emerging analysis could address gaps in the literature and/or contribute to theory in relation to immigrant health disparities.

### Policy analysis

We will document policy issues project-wide from the observational and meeting logs using the *Center for Disease Control’s (CDCs) Policy Analytic Framework* [126] to inform our future implementation of this model of peer support in the context of a larger multi-level intervention. We will identify problems faced by participants, appropriate policy solutions/options, and outline strategies for policy change.

## Discussion

The Tertulias research focuses on mental and behavioral health issues and there is a risk that we could identify a participant who needs support or assistance beyond the regular components of participation in a study and because participants in this study are from a “hard-to-reach” population that could cause recruitment and retention issues. To address these concerns, we have taken various measures to ensure patient safety and data quality, including the creation of two bodies:

- **■**) The Tertulias Community Advisory Board (CB):
  - **CB purpose. **The purpose of the Tertulias Community Advisory Board (CB) is to provide community stakeholder input to ensure the cultural competence and contextual appropriateness of the research. The CB will operate by consensus to make recommendations to the research team regarding project operations and provide input for interpretation of findings.
  - **CB composition**. The CB will be composed of 6 women who have been or are part of the original Tertulias group at the Hopkins Center and who participated in the preliminary research for the proposed Tertulias research study. Select Tertulias research team members will attend to provide information about the research and project progress and to obtain CB input.
  - **CB operations.** The CB will meet quarterly at a time convenient to CB members at the facility of our partner organization, the Hopkins Center for Children and Families, or if necessary because of COVID restrictions, by Zoom. CB meetings will be conducted in Spanish. All CB attendees (external and from the team) will be fluent in Spanish. CB members will receive a $250 stipend for attending each meeting. Notes will be taken to document input, suggestions, discussion points, interpretations, decisions, and action steps.
  - **CB disclosures/commitments**. All CB members will provide signed confirmation of no conflict of interest with this study prior to the first CB meeting. All CB members will also sign a confidentiality statement promising not to disclose deliberations of the CB.
  - **CB process. **During the first meeting, the CB will create a charter defining the logistics and functioning of CB meetings. The charter will specify: meeting dates/times, format for presentation of data and discussion, and other issues relevant to committee operations. The CB Meetings will be co-facilitated by PI Page-Reeves and Co-Is J. Perez and Regino. At CB meetings, the PI and other research team members will report on project progress and challenges. The CB members will provide input and assist with interpretation of findings.
  - **CB Scope: **The CB will provide input regarding:
  1. **Project Cultural Competence.** The CB will assess cultural competence of study procedures and methods. The CB will review all study documentation such as the recruitment flyer, the recruitment script, the consent form, data collection instruments, and study procedures to ensure that the information is being gathered in a culturally competent fashion.
  2. **Study Recruitment, Enrollment, and Attrition Data.** The CB will be attentive to slow recruitment and enrollment, low participation, or high attrition. The CB will review the number of participants recruited, number who decline to participate, the number screened, the number who do not qualify, the number of enrolled in the study, the number who leave the study and when they leave the study, and detail of reasons for each category. We will create a detailed Consort Diagram to depict these components of the study. The Consort Diagram will be shared with the CB.
  3. **Recommendations for Improvement.** The CB will make recommendations for improving performance of the study. Recommendations from the CB will be implemented by the research team following CB meetings.
  4. **Interpretation of Findings.** The CB will be presented with preliminary findings from Research Team analysis of data. The CB will provide input for interpreting these findings. Input from the CB will be incorporated into final analyses.
- **■**) The Tertulias Data and Safety Management Committee (DSMB):
  - **DSMB purpose. T**he purpose of the Tertulias Data & Safety Monitoring Board (DSMB) is to provide oversight for ensuring participant safety and for monitoring the study for rigorous scientific conduct.
  - **DSMB operations.** DSMB Membership is detailed below. The DSMB will meet quarterly or as necessary at the UNM Office for Community Health. DSMB meetings will be conducted in English. Notes will be taken during the meetings to capture discussion points, decisions, and action items.
  - **DSMB disclosures/commitments.** All board members will provide signed confirmation of no conflict of interest with this study prior to the first DSMB meeting. All board members will also sign a confidentiality statement promising not to disclose deliberations of the DSMB.
  - **DSMB process.** During the second meeting, the DSMB will create a charter defining the logistics and functioning of DSMB meetings. At DSMB meetings, the PI and other research team members will report on project progress and challenges. The Chair will lead the DSMB discussion. The DSMB will follow these decision-making procedures:  DSMB meetings require a quorum. A quorum is defined as three DSMB voting members.  A simple majority [3] is required to pass a motion. If all DSMB voting members are not present for important regulatory document approval, the Chair will use electronic voting +/- 24 hours before and after the meeting.
- requires a closed meeting, research team members will not attend. However, the DSMB may decide at any time to close the meeting. Voting on recommendations will follow Roberts’ Rules of Order. Minutes of DSMB meetings will be kept. If concerns are identified, the report will outline the concerns, the board’s discussion of the concerns, and the basis for any recommendations that the DSMB has made in response to the concerns. The DSMB will plan to meet quarterly.
  - **DSMB scope. The DSMB will monitor:**
    This is a low risk study, so there are no safety endpoints that would result in early termination of the study. However, because this research focuses on mental and behavioral health issues, there is a risk that we could identify a participant who needs support or assistance beyond the regular components of participation in the study and because participants in this study are from a “hard-to-reach” population that could cause recruitment and retention issues. DSMB meetings will generally be open, with research team members attending to provide information and updates. If the DSMB.

1. **Participant Safety Data**. The DSMB will review protocols and resources for patient referral in the event of a mental or behavioral health emergency associated with participation in the research (e.g., if a woman reports domestic violence or extreme depression/suicidality during a group discussion of in the context of a data collection appointment). The DSMB will evaluate potential or actual patient safety issues and ensure that patient safety is sufficiently being addressed. The DSMB will be able to review individual participant data as appropriate and will review summaries of all adverse events. Serious Adverse Events (SAEs) will be reviewed individually and in detail.
2. **Protocol Compliance.** The DSMB will assess compliance with the protocol, including reports of protocol deviation and compliance with safety and administrative procedures. The DSMB will be able to review all study documentation such as the research protocol, the recruitment flyer, the recruitment script, the consent form, data collection instruments, and study procedures to ensure that the information being gathered is sufficient for patient safety and for scientific rigor of the study as outlined in the protocol. The DSMB may also monitor the quality and completeness of the study data being collected, including missing or erroneous data.
3. **Study Recruitment, Enrollment and Attrition Data.** The DSMB will be attentive to slow recruitment and enrollment, low participation, or high attrition. The DSMB will review the number of participants recruited, number who decline to participate, the number screened, the number who do not quality, the number of enrolled in the study, the number who leave the study and when they leave the study, and detail of reasons for each category. The PI will create a detailed Consort Diagram to depict these components of the study. The Consort Diagram will be shared with the DSMB.
4. **Recommendations for Improvement.** The DSMB will make recommendations for improving performance of the study. Recommendations from the DSMB will be implemented by the research team following DSMB meetings.
5. **Responsibility of the Principal Investigator (PI):** The PI will prepare reports summarizing the above materials for review by the DSMB in advance of each meeting.

- **DSMB composition**. The DSMB is composed of five individuals. The Chair for the DSMB and will lead DSMB meetings with the four other members. The DSMB’s five voting members are external to the research team. Composition of the DSMB is diverse to reflect the nature and theme of the proposed study. The term of service on the DSMB will be for the duration of the project. Select Tertulias research team members may attend to provide information about the research and project progress. Only the five DSMB members will be have the ability to vote.

### Ethics

#### Approvals

This study was approved by the Human Research Protections Office (HRPO) of the University of New Mexico on September 18, 2019 (#19–160). Written consent will be obtained whenever possible, but we obtained permission for oral consent when necessary as described 2 paragraphs below in the Consent Section.

#### Protocol amendments

Significant protocol modifications will be reported to ClinicalTrials.gov.

#### Consent

We obtained a waiver of written consent for gathering the data required to determine eligibility and contact information. This information is necessary up front for us to recruit because we will be conducting group informational interest meetings describing the research and the consent form, and because this is a randomized study, we need to show potential participants the randomization video to determine if they really do want to participate. We will destroy the contact information from participants who later decline to participate at the time of written consent.

All participants who enter the study will provide informed consent. We obtained permission to obtain oral consent from those who are unable to attend an in-person baseline data collection appointment because of COVID-19 restrictions. When we are attempting to schedule the appointment, it may not be feasible to have the data collectors meet with the participant in-person where the consent form would be signed prior to answering baseline survey questions or obtaining the baseline hair sample if the participant answers affirmatively to COVID-19 screening questions (e.g., positive COVID-19 test, contact with a COVID-19 person, recent out-of-state travel, fever or other symptoms of illness). We will attempt to conduct in-person data collection appointments because we need a baseline hair sample in addition to the survey questions. However, if the participant is unable to meet in-person, we do not wish to exclude the participant since the intervention is likely being conducted by Zoom and there is no further risk of contagion apart from the baseline data collection appointment. Therefore, we will obtain oral consent by phone or Zoom and document the participant’s name, the date of consent and the team member who conducted the consent. All of the consenting participants will have attended a two-hour Zoom meeting where we describe the study and the contents of the consent form in detail and show the randomization video. Following oral consent, we will provide the participant with a printed copy of the consent form by mail. We will conduct the baseline survey by phone or Zoom and we will attempt to schedule the hair sample collection at a later date, if feasible. If not feasible, the lack of hair sample for that participant will be recorded as missing data.

#### Confidentiality

Guidelines for the protection of participant privacy and confidentiality will be followed in all cases. All members of the research team will maintain current Human Subjects training. They will understand the importance of privacy issues and their responsibility to maintain the highest research ethical standards in all respects. As indicated above, we obtained permission to make alterations to the consent process using a meeting, a PowerPoint presentation and a video. We believe that this will lead to superior understanding by the participants regarding the nature of participation and the concept of randomization. It will also make the study more feasible. However, women who choose will be able to have the information presented individually in a private setting. Also, we obtained permission to obtain oral consent from those who are unable to attend an in-person baseline data collection appointment because of COVID-19 restrictions. When we are attempting to schedule the appointment, it may not be feasible to have the data collectors meet with the participant in-person where the consent form would be signed prior to answering baseline survey questions or obtaining the baseline hair sample if the participant answers affirmatively to COVID-19 screening questions (e.g., positive COVID-19 test, contact with a COVID-19 person, recent out-of-state travel, fever or other symptoms of illness). We are attempting to conduct in-person data collection appointments because we need a baseline hair sample in addition to the survey questions. However, if the participant is unable to meet in-person, we do not wish to exclude the participant since the intervention is being conducted by Zoom and there is no further risk of contagion apart from the baseline data collection approintment. Therefore, we will obtain oral consent by phone or Zoom and document the participant’s name, the date of consent and the team member who conducted the consent. All of the consenting participants will have attended a two-hour Zoom meeting where we describe the study and the contents of the consent form in detail and show the randomization video. Following oral consent, we will provide the participant with a printed copy of the consent form by mail. We will conduct the baseline survey by phone or Zoom and we will attempt to schedule the hair sample collection at a later date, if feasible. If not feasible, the lack of hair sample for that participant will be recorded as missing data.

Surveys, gathering of hair samples, and interviews will be conducted at a location to provide privacy. The final group session will not be private as it will be a group discussion resembling a focus group.

At the beginning of each 12-month group cohort, the facilitator will instruct participants regarding privacy measures for the group context. The group participants will discuss the concept of privacy and if there is consensus, they will sign a letter committing not to discuss the content of the sessions with outsiders. All participants will be asked to sign a receipt for a merchandise card incentive which will be used for project accounting purposes only and will not be linked with or associated with research data.

Consents and participant contact information sheets will be assigned a study ID. The ID and the contact sheet will be the only link between the participant and the data. We need to be able to maintain a link between the participant and the data to be able to notify participants of meeting logistics, to schedule follow-up appointments at 12 months, to schedule interviews, and to be able to make bi-monthly check-in calls with control participants. Consent Forms, the list of participants who provided oral consent, and contact information will be stored in separate locked cabinets in the office of the PI and with access only by the investigators. All other research data will be de-identified. The only link between the participant and the data or contact information will be through the study ID. Contact information will be destroyed 5 years following the end of the study. Consent Forms and the list of those who provided oral consent will be kept for 3 years following the end of the project, at which time they will be destroyed.

#### Declaration of interests

None of the authors have any financial or other conflicts of interest to report.

#### Access to data

Participant contact information will be considered confidential and will not be shared. De-identified quantitative project data will be shared per our Data Sharing Protocol. Qualitative data cannot be de-identified and therefore will not be shared.

### Dissemination Plan

1. We will present findings from this study at the New Mexico Public Health Association (NMPHA) which is held annually in either Albuquerque or Las Cruces. We have budgeted for us to be able to present our work as a panel involving multiple members of the team, including community partners and members of the Advisory Board. We believe that this work will be of great interest to the professional community in New Mexico as the issues of social isolation and depression among FMIs are recognized as key health disparities. Our ability to demonstrate a scientific and measurable quantitative impact will be received as extremely exciting.
2. Members of our team from UNM, Centro Sávila and One Hope Centro de Vida will be available to advise organizations interested in pursuing this model.
3. We recognize that it is essential that we do not just go into the community and create something and then leave the participants in a lurch when the research is done. Therefore, we have created an infrastructure to ensure that the participants have the possibility of continued social support. Centro Sávila has a continuous supply of interns (social work and counseling students) who spend at least 1 year working with clients about mental and behavioral health issues. When Tertulias groups end, we will invite participants to continue meeting with facilitation by a pair of Centro Sávila interns. This infrastructure creates a feasible way to create continuity and sustainability. One Hope will seek funding to be able to support the facilitators for groups held at their site.
4. We will present findings from this study at national professional conferences such as the American Public Health Association, the American Anthropological Association, the Health Disparities Conference, the Addressing Health Disparities Conference, the Southwest Anthropological Association Conference, or other relevant venues.
5. We will publish findings from this study in peer-reviewed journals (Table 1).

**Table 1:** SPIRIT Flow Diagram^*^

ACTIVITY	Study Period																
	Enrollment	Allocation	Month														Close-out
TIMEPOINT	-2	-1	0	1	2	3	4	5	6	7	8	9	10	11	12	13	13
Enrollment																	
Eligibility Screen	X																
Informed Consent	X																
Allocation		X															
Intervention																	
Arm 1: Intervention weekly peer group meetings				X	X	X	X	X	X	X	X	X	X	X	X		x
Arm 2: Control Placebo Phone Call					X		X		X		X		X		X		x
Assessments																	
Baseline Survey & Hair Sample			X														
Post Survey & Hair Sample																X	
Meeting Logs, Policy Log, and Observational Notes				X	X	X	X	X	X	X	X	X	X	X	X		
Interviews (Y2 & Y3 only)					X	X	X										
Participant Creative Project													X				
Group Session															X		

^*^We will replicate these activities four times over 4 years

## Supplementary Information

**Additional file 1.**

## Data Availability

De-identified quantitative datasets (survey and hair cortisol results) used and/or analyzed during the current study will be available from the corresponding author on reasonable request. Qualitative data will not be shared because it will contain individually identifiable information.

## Abbreviations

FMI: Female Mexican immigrant
UNM: University of New Mexico
NIMHD: National Institute of Minority Health and Health Disparities
PIRE: Pacific Institute for Research and Evaluation
One Hope: One Hope Centro de Vida Health Center
CES-D: Center for Epidemiologic Studies Depression Scale (109, 110)
CD-RISC 25: Connor-Davidson Resilience Scale− 25 (112)
MOS-SSS: Social Support Scale (113, 114)
PSS − 14: Perceived Stress Scale-14 (111)
TIP Scale: Trauma-Informed Practice Scale (115, 116)
