# Supplementary material for: A randomized control trial to test a peer support group approach for reducing social isolation and depression among female Mexican immigrants
Source: BMC Public Health. 2021 Jan 11;21:119. doi: 10.1186/s12889-020-09867-z (PMC7798010; doi:10.1186/s12889-020-09867-z)
Supplement: Supplementary file 1 — Additional file 1. [file 12889_2020_9867_MOESM1_ESM.docx]

**The University of New Mexico Health Sciences Center**

**Consent and Authorization to Participate in a Research Study**

**KEY INFORMATION FOR PARTICIPANTS***.*

**Version** 12-17-19

You are being invited to take part in a research study about women immigrants born in Mexico and how their social relationships impact health.

**WHAT ARE THE PURPOSE, PROCEDURES, AND DURATION OF THE STUDY?**

We will recruit 240 female immigrants from Mexico. Half will be part of a women’s group that will meet weekly for a year. The other half will receive a call from a researcher to check-in every other month, but will not be part of a group. All participants will answer some survey questions and be asked to provide a small sample of their hair at the beginning and end of the 12 months. A few will be asked to also answer some more detailed questions in an interview.

By doing this study, we hope to learn if a women’s group is something that helps immigrant women make friends, get advice from other women, learn how to find resources, feel less isolated and depressed, and be more empowered. Your participation in this research will last 12 months.

**WHAT ARE THE KEY REASONS I MIGHT CHOOSE TO VOLUNTEER FOR THIS STUDY?**

We have found that immigrant women from Mexico often have a hard time making friends and end up feeling depressed and isolated. Being part of the women’s group in this project may help with these problems, but may also be a safe space to talk about feelings, emotions and experiences.

**WHAT ARE THE KEY REASONS I MIGHT CHOOSE NOT TO VOLUNTEER FOR THIS STUDY?**

Some women may not want or be able to participate because they don’t have time, or because they don’t like the idea of being in a women’s group.

**DO I HAVE TO TAKE PART IN THE STUDY?**

No. Participation in this study is voluntary.

**WHAT IF I HAVE QUESTIONS, SUGGESTIONS, OR CONCERNS?**

The person in charge of this study is Janet Page-Reeves, a researcher at the University of New Mexico (UNM). If you have questions, suggestions, or concerns regarding this study or you want to withdraw from the study, you can call her at 505-306-3041 or email to [JPage-Reeves@salud.unm.edu](mailto:JPage-Reeves@salud.unm.edu)

If you have any questions or concerns about your rights as a participant in this research, contact staff in the University of New Mexico Health Sciences (UNMHSC) Human Research Review Committee (HRRC) between the business hours of 8AM and 5PM, Mountain Standard Time (MST), Monday-Friday at 505-272-1129.

**DETAILED CONSENT**

**ARE THERE REASONS WHY I WOULD NOT QUALIFY FOR THIS STUDY?**

You will not qualify if you:

- Are not female
- Were not born in Mexico
- Are under the age of 18
- Are not fluent in Spanish
- Do not have income above 250% of the Federal Poverty Level
- Are currently incarcerated

**WHERE IS THE STUDY GOING TO TAKE PLACE AND HOW LONG WILL IT LAST?**

Your participation in this study will last 1 year.

The study includes different parts:

- **Appointments to complete the 2 surveys and give the 2 hair samples** will be at one of our partner sites (One Hope Clinic or Centro Savila) or another location convenient for you, if it is feasible.
- **If you are in Group 1**, then you will not have any other research activities, but you will receive a check-in call from one of our staff every other month.
- **If you are in Group 2**:
  - You will attend a weekly women’s group meeting at either One Hope Clinic or Centro Savila.
  - We may invite you to do an interview at one of our partner sites (One Hope Clinic or Centro Savila) or another location convenient for you, if it is feasible.
  - We will use the last group meeting as a research evaluation discussion session.

**WHAT WILL I BE ASKED TO DO?**

**Today you are being asked to sign the consent form**.

We showed you a video about “randomization.” **After you sign the consent form, we will let you know which group you are in. You will receive $50 for participating in the randomization meeting.**

**All participants in this study will be asked to take a survey and give a sample of hair at two time points 12 months apart.**

- For participating in this study, all participants will receive a total of $200 in Walmart Cards ($50 each time they take the survey and $50 each time they provide a hair sample). Some participants will be invited to do an interview and they will also receive a $50 Walmart card. Some participants will participate in a discussion session and will also receive a $50 Walmart card.
- You will receive the card if you participate in a research activity even if you have to leave early or if you decide to stop in the middle.
- We will ask you to sign a receipt for the card for our financial record files.

**Today, after you sign the consent form and are placed in a group, we will set up an appointment to ask you survey questions and take a small sample of your hair**.

- **Survey -** The survey will include question including your age or the number of children you have, and your experiences.
- **Hair –** We will collect a small sample of your hair to measure it for chronic stress. We know that stress really influences people’s health. If you are under stress all the time, we call this chronic stress. Having chronic stress may lead your body to release a hormone called cortisol.

**If you are placed in Group 1**:

- You will receive a phone call every 2 months to update your contact information and also just to check-in with you.
- We ask that Group 1 participants notify us if they have a change of phone number or address.
- We will schedule a second appointment with you at the end of the 12 months to re-take the survey and give another hair sample.

**If you are placed in Group 2**:

- You will attend a weekly women’s group (at either One Hope Clinic or Centro Savila) with about 5-10 other women.
- The meeting will last about two hours.
- We will provide free babysitting if you need it.
- For the first meeting, we will provide a meal, but after that, it will be potluck style.
- The meeting will be conducted by two facilitators **in Spanish** and the women in the group will decide what they want to talk about.
- A member of the research team will occasionally attend the meetings and take notes.
- Later in the year, the group will create a group binder where you can contribute something creative that you make—like a drawing or painting, a poem or story you write, a recipe, or a letter to the other participants telling them about what this group means to you. You can really contribute whatever you want to the binder. We will make copies of the binder so that each member of the group is able to keep one.
- The last group session at the end of the 12 months will be a group discussion. You can share how you feel about it and if it is something that is of valuable to you. Because this last session is research rather than a peer group sessions, you will receive a $50 Walmart card for participating.
- In addition, we will invite a few women to do an interview where we will ask more in-depth questions. Women who do the interview will also get a $50 Walmart card.
- For the last session meeting and each of the interviews, we will make an audio recording and have it transcribed so that we can see what people said on paper—that is easier than having to listen to it repeatedly. After we make the transcription, we will destroy the audio recording.
- At the end of the 12 months, we will schedule another appointment with you to take the survey again and give another hair sample. Again, for each of those, you will receive a $50 Walmart card.

**WHAT ARE THE POSSIBLE RISKS AND DISCOMFORTS?**

Participation in this study presents minimal risk and we do not think that you will experience any negative consequences or effects. However, some people feel anxiety, stress, or, inconvenience when they answer questions about themselves or talk about their experiences. In addition, although we go to great lengths to keep everything confidential, there is always the possibility of a loss of privacy. As a precaution against this, we go further than just keeping your information stored safely. We also get something called “a Certificate of Confidentiality” that allows us to be able to keep your name and information private. In addition to these risks, there may be some risk that we cannot anticipate.

**WILL I BENEFIT FROM TAKING PART IN THIS STUDY?**

We anticipate that this project will have a positive impact in the lives of participants. Furthermore, this research will contribute to the science of addressing health disparities for among Mexican immigrant women which may be important to you in terms of helping others.

**WHAT WILL IT COST ME TO PARTICIPATE?**

There are no financial costs associated with taking part in the study. You may incur some expense to travel to meetings or appointments, and if you are in Group 2, you will be asked to be on a schedule to occasionally bring a food dish in potluck style to some of the meetings.

**WHO WILL SEE THE INFORMATION THAT I GIVE?**

We will not share your name or contact information with anyone. When we analyze the results of this study, we will share the findings with people through presentations at professional conferences and in written articles, but no one will be able to know that you participated because we will combine the information you provide with that we gather from other women. After the project is over, we may share the information that we gather with others, but what we share will not contain your name or contact information or be in any way connected to you.

We will only keep a record of your name on the consent form and your contact information to schedule appointments or to communicate with you regarding the details of group meetings.

We will store the consent forms and contact sheets in separate locked cabinets. We store the information gathered in this project in a secure database.

We will do everything we can to protect the privacy of all your personal information, but we cannot guarantee confidentiality 100%. All data and hair samples from this study will be assigned a number and we will not use your nam. The UNM Human Research Protections Office (HRPO) that makes sure we are treating people who participate in research at UNM properly sometimes asks to see our records—not to identify you, personally, but to see how we are doing our research. However, your name will not be used in any published reports, publications or presentations about this study.

We will obtain a Certificate of Confidentiality for this project from the National Institutes of Health. This allows researchers to refuse to disclose your identity. A Certificate of Confidentiality does not prevent you or a member of your family from voluntarily releasing information about yourself or your involvement in this research. We may be required to show your information to other people if the funder want to evaluate portions of our project or if we become aware that a participant has committed a serious crime such as child abuse. For example, the law may require us to share your information with the responsible agency for the following reasons:

- If you report information about a child being abused.
- If you pose a danger to yourself or someone else.
- If you have a reportable disease or condition.

**CAN I CHOOSE TO WITHDRAW FROM THE STUDY EARLY?**

Yes, you may do so at any time. If you choose to leave early, the data you have already provided will still be used in the study. Even if you cannot participate, it is helpful to us to still keep the information you have already provided in the study unless you notify us in writing by sending a letter to:

Janet Page-Reeves

MSC 09 5065

1 University of New Mexico

Albuquerque New Mexico 87131

Please note that the research team will not destroy information you have provided that has already been used or analyzed or shared before the date that your letter is received. That information will not be able to be removed.

Occasionally investigators conducting the study need to withdraw a participant.

This would only happen:

- If you are disruptive during group sessions or have a conflict with another participant.
- If we have to stop the entire project for some unforeseen reason

**WILL I BE PAID FOR PARTICIPATING IN THIS STUDY?**

Participants in both Group 1 and Group 2 will receive a total of $200 for taking two surveys and providing two hair samples ($50 Walmart card for survey and each hair sample). If you are in Group 2, you will also receive a $50 Walmart card if you are asked to participate in an interview, and another for the final group session.

Apart from the last meeting described above, you will not receive payment for participating in the other group meetings or check-in phone calls.

**FUTURE USE OF MY NAME AND CONTACT INFORMATION**

We will not share your personal information with anyone outside of our study.

However, Janet Page-Reeves, the director of this study, will keep your name and contact information on a list called a contact list. That way, if she conducts another study in the future that she thinks you might be interested in or qualify for, we can contact you. **See the** **Appendix below called Subject Information to be stored for future research for more information.**

**HIPAA AUTHORIZATION FOR USE AND DISCLOSURE OF MY PROTECTED HEALTH INFORMATION (PHI).**

As part of this study, we will be collecting protected information about you such as your name, your contact information, and answers to questions about your health. This information is “protected” because it is identifiable or “linked” to you.

**Protected Health Information (PHI)**

By signing this Consent Document, as described in this consent form, you are allowing the investigators to use your protected health information for the purposes of this study. This information includes your name, date of birth, age, the answers you provide to some of the survey questions, and the hair samples.

In addition to researchers and staff at UNMHS and other groups listed in this form, there is a chance that your health information could be shared (re-disclosed) outside of the research study and no longer be protected by federal privacy laws. Examples of this include health oversight activities and public health measures, and safety monitors.

**Right to Withdraw Your Authorization**

Your authorization for the use and disclosure of your health information for this study shall not expire unless you cancel this authorization. Your health information will be used or disclosed as long as it is needed for this study. However, you may withdraw your authorization at any time provided you notify the UNM investigators in writing. To do this, please send letter notifying them of your withdrawal to:

Janet Page-Reeves

MSC 09 5065

1 University of New Mexico

Albuquerque New Mexico 87131

Please be aware that the research team will not be required to destroy or retrieve any of your health information that has already been used or shared before the date that your withdrawal is received.

**WHO CAN I CALL IF I HAVE QUESTIONS ABOUT THIS STUDY?**

If you have any questions or concerns at any time about the research study, Dr. Janet Page-Reeves will be glad to answer them at 505-306-3041. If you would like to speak with someone other than Dr. Page-Reeves, you may call the UNMHSC HRRC at (505) 272-1129.

**WHO CAN I CALL IF I HAVE QUESTIONS ABOUT MY RIGHTS AS A RESEARCH PARTICIPANT?**

If you have questions regarding your rights as a research participant, you may call the UNMHSC HRRC at (505) 272-1129. The HRRC is a group of people from UNM and the community who are not involved in this project. They look out for the interests of people who participate in research projects at UNM. For more information, you may also check out the HRRC website at <https://hsc.unm.edu/research/hrpo/>

**INFORMED CONSENT SIGNATURE PAGE**

You are participating. This consent includes the following:

- First page with key information
- Detailed Consent

You will receive a copy of this consent form after it has been signed.

**Signature of research participant**  **Date**

**Printed name of research participant**

**Signature of researcher**  **Date**

**Printed name of researcher**

**The University of New Mexico Health Sciences Center**

**APPENDIX**

**Participant Information to be Stored for Future Research**

**WHAT INFORMATION THAT I PROVIDE WILL BE STORED?**

- We will store a contact list.
- We will also store the information that we gather during the study.

**WHAT IS A CONTACT LIST AND WHAT IS THE PURPOSE OF THIS LIST?**

- It is a list of people’s names who participated in the study and their contact information.
- **The purpose of this contact list** is to be able to contact people to see if they want to participate in another study that we do in the future.
- We plan to invite all 240 participants of this study to be in our contact list.

**WHAT IS THE OTHER INFORMATION THAT WILL BE STORED?**

- Answers you gave us to survey questions and interviews
- Transcripts from the discussion during the final group session
- Notes taken by the researchers
- Results of the cortisol testing.
- **This information will not include your name and will be made anonymous when it is stored** so it will no longer be connected to you.

**WHERE WILL INFORMATION BE STORED AND FOR HOW LONG?**

- The contact list will be stored in the office of Janet Page-Reeves, the project director at UNM .
- The other information that you provide will be stored in a secure UNM data base.
- We will keep this list and the other information for 5 years and then it will be destroyed.

**HOW WILL THE CONTACT LIST INFORMATION BE SHARED WITH OTHER INVESTIGATORS?**

- We will not share this contact list with anyone else.
- Only Dr. Page-Reeves will be able to use it to invite people to participate in future studies.

**HOW WILL THE OTHER INFORMATION BE SHARED WITH OTHER INVESTIGATORS?**

- We will only share the anonymous survey information or cortisol testing results with researchers if they ask for it and are able to show that they will use it properly. They will have to sign some documents that will be required by UNM.
- We will not share the transcripts of interviews or group sessions, or the notes taken by the researchers because these might contain information that could identify a participant.
- This information will be stored in the office of Dr. Page-Reeves and she will not share it with other researchers.
- After 5 years, this information will also be destroyed.

**HOW IS MY PRIVACY AND CONFIDENTIALITY PROTECTED?**

- We will take careful steps to keep your information confidential. Dr. Page-Reeves will store the list on a UNM password-protected computer and only study team members will have access to it.
- There is a risk that someone might get access to the information stored in the contact list. In spite of the security measures and safeguards we will use, we cannot guarantee that your identity as a research participant will never become known.

**DOES TAKING PART IN THE CONTACT LIST COST ANYTHING?**

- There will be no financial costs to you for your name and contact information to be in the contact list.

**WHAT IF I CHOOSE NOT TO PARTICIPATE OR CHANGE MY MIND AND WANT TO WITHDRAW FROM TAKING PART IN THE CONTACT LIST?**

- Taking part in the contact list is optional and voluntary. You may withdraw your permission to continue taking part in the list at any time. To do so, you must send a written withdraw request to Janet Page-Reeves at

[JPage-Reeves@salud.unm.edu](mailto:JPage-Reeves@salud.unm.edu). She will destroy your information that has been stored.

**FUTURE USE AUTHORIZATION**

- Yes, I choose to participate in the optional contact list. ____________Initials
- No, I choose not to participate in the optional contact list. ___________Initials
